# Early sleep after action observation and motor imagery training boosts improvements in manual dexterity

**DOI:** 10.1038/s41598-023-29820-5

**Published:** 2023-02-14

**Authors:** Federico Temporiti, Alessandra Calcagno, Stefania Coelli, Giorgia Marino, Roberto Gatti, Anna Maria Bianchi, Manuela Galli

**Affiliations:** 1 Physiotherapy Unit, Humanitas Clinical and Research Center - IRCCS, Via Manzoni 56, Rozzano, Milan Italy; 2 Department of Electronic, Information and Bioengineering, Politecnico Di Milano, Via Ponzio 34, Milan, Italy; 3 Department of Biomedical Sciences, Humanitas University, Via Rita Levi Montalcini 4, Pieve Emanuele, Milan, Italy

**Keywords:** Sensorimotor processing, Motor control

## Abstract

The systematic observation and imagination of actions promotes acquisition of motor skills. Furthermore, studies demonstrated that early sleep after practice enhances motor learning through an offline stabilization process. Here, we investigated behavioral effects and neurodynamical correlates of early sleep after action observation and motor imagery training (AO + MI-training) on motor learning in terms of manual dexterity. Forty-five healthy participants were randomized into three groups receiving a 3 week intervention consisting of AO + MI-training immediately before sleeping or AO + MI-training at least 12 h before sleeping or a control stimulation. AO + MI-training implied the observation and motor imagery of transitive manual dexterity tasks, whereas the control stimulation consisted of landscape video-clips observation. Manual dexterity was assessed using functional tests, kinematic and neurophysiological outcomes before and after the training and at 1-month follow-up. AO + MI-training improved manual dexterity, but subjects performing AO + MI-training followed by early sleep had significantly larger improvements than those undergoing the same training at least 12 h before sleeping. Behavioral findings were supported by neurodynamical correlates during motor performance and additional sleep-dependent benefits were also detected at 1 month follow-up. These findings introduce a new approach to enhance the acquisition of new motor skills or facilitate recovery in patients with motor impairments.

## Introduction

The observation of actions activates neural structures involved in the execution of observed tasks through a mirror mechanism^1^. The Mirror Neuron System (MNS) is a frontoparietal brain network responsible for the mirror mechanism, which allows the central nervous system to encode visual inputs into a motor representation of observed actions^2^. This process enables action understanding and facilitates motor learning through the building of a motor memory^2–4^. The aforementioned MNS peculiarities enabled the development of the action observation (AO) training to improve motor skills in healthy subjects or patients with motor and functional impairments, as well as to prevent the decline of motor performance induced by immobilization^5,6^. This rehabilitative approach consists of asking subjects to watch video-clips including motor contents. AO can be followed by motor imagery (MI) of observed tasks, leading to the implementation of training including both AO and MI components (AO + MI-training)^2^. In fact, MI performed immediately after or during the observation of a motor task further improves motor learning, since a partial overlap in terms of brain activations has been described during observation and imagination of a motor task^7,8^. Consistently, studies demonstrated that AO + MI produces higher activity in motor brain areas compared to MI or AO alone^9,10^. Furthermore, the characteristics of the visual stimuli (e.g., person-related and viewing perspective and task transitivity) and the familiarity of observed and imagined actions with the personal motor repertoire also play a role in modulating brain activity and determining AO + MI-training efficacy^11^.

Manual dexterity is the ability to generate voluntary, fine and coordinated movements for grasping and manipulating objects^12^. This skill has been reported as improvable using AO/AO + MI-training in healthy subjects and patients with neurological disorders^5^. Rocca and co-workers found increased hand performance after a 2-week AO-training in healthy subjects, and improvements were accompanied by grey matter structural changes in MNS brain areas^13^. Moreover, Bek and co-workers demonstrated that the imitation of a hand dexterity task resulted more accurate when observation was followed by MI^14^. Recovery of manual dexterity induced by AO/AO + MI interventions has been also demonstrated in patients with subacute and chronic stroke and in subjects with multiple sclerosis^5,9^. In patients with stroke, benefits were supported by neurophysiological data revealing decreased activity of frontoparietal sensorimotor areas during a hand dexterity task as a result of motor learning^15^. In addition, activity of MNS regions involved in motor learning (e.g., ventral premotor and inferior parietal areas) increased^15^. Furthermore, Sun and co-workers demonstrated that a 4-week AO + MI-training increased hand dexterity in patients with stroke, where synchronous AO + MI led to greater improvements and sensorimotor cortex activations than asynchronous AO + MI^16^. Similarly, hand function improvements in patients with multiple sclerosis occurred with structural changes in frontotemporal areas and increased MNS recruitment during a manipulation task^17^.

When considering strategies for enhancing motor learning, a key role of sleep has been documented on motor memory formation and consolidation after a motor training^18,19^. Motor learning implies the storage of motor traces into a motor memory, which allows for their retrieval during motor execution^20^. In this scenario, sleep has been reported to ensure the long-lasting storage of newly acquired motor traces through an offline stabilization process after the training^21^. In particular, sleep beneficial effects have been reported in healthy subjects and patients with central nervous system damage, where the closeness of sleep occurrence to practice seems to enhance motor learning^18^. In addition, a sleep-dependance of motor learning has been documented by neuronal activity patterns occurring in specific sleep phases, such as sleep spindles during the non-Rapid Eye Movement (REM) sleep (stage 2) and Slow Wave Sleep^22^. These electrophysiological events have been considered as associated with sleep-dependent motor memory consolidation^23,24^.

Based on the current evidence on AO/AO + MI effects on motor performance and sleep advantages in enhancing the acquisition of motor skills, Van Der Werf and co-workers investigated sleep effects on motor learning induced by observation in healthy subjects. These results demonstrated that the execution of a finger tapping sequence was more accurate when subjects underwent a sleep session within 12 h after the observation^25^. Moreover, a more recent study showed that 4 weeks of AO followed by immediate sleep after each training session enhanced upper limb recovery in patients with stroke. However, a non-randomized design was adopted, AO included a single motor task and MI was not required to participants^26^. Furthermore, longitudinal changes in terms of cortical activity have never been investigated in the aforementioned studies.

Against this background, it is reasonable to speculate that sleep might increase learning of trained motor skills such as manual dexterity, when occurring immediately after AO + MI-training. Therefore, the study was aimed at investigating the behavioral effects and neurodynamic correlates of AO + MI-training followed by immediate sleep on manual dexterity in healthy subjects. To this end, healthy participants were randomized into three groups receiving a 3-week intervention consisting of AO + MI-training immediately before sleeping (AOMI-sleep) or AO + MI-training at least 12 h before sleeping (AOMI-control) or a control stimulation (Control). Manual dexterity was assessed using functional tests (Purdue Pegboard Test—PPT and Finger Tapping Test—FTT), kinematic (kinematic indexes during Nine Hole Peg Test—NHPT) and neurophysiological outcomes (electroencephalographic signals recording during NHPT) before (T0) and after the training (T1) and at 1-month follow-up (T2). In the case of positive findings, the use of AO + MIfollowed by an early sleep-window may be considered to further enhance the acquisition of new motor skills in healthy subjects or facilitate functional recovery in patients with motor impairments.

## Results

None of the participants withdrew from the study and no between-group differences were found for baseline characteristics, sleep quality assessed using the Pittsburg Quality Sleep Index (PSQI) and number of sleep hours per night during the training period (Table 1).

**Table 1:** Characteristics of study participants. Data are shown as mean and standard deviation.

	AOMI-sleep	AOMI-control	Control	*p*-value
Age [y]	24.1 ± 2.9	22.9 ± 2.1	23.3 ± 2.4	0.377
Gender [M/F]	6/9	9/6	9/6	0.448
Height [cm]	171 ± 10	177 ± 10	176 ± 11	0.28
Weight [kg]	63.7 ± 10.9	66.6 ± 12.0	71.8 ± 20.9	0.347
PSQI [points]	4.3 ± 2.8	4.9 ± 2.6	3.1 ± 1.4	0.133
Sleep hours per night [n]	7.9 ± 0.9	7.6 ± 0.8	8.1 ± 0.4	0.092

AOMI: Action Observation plus Motor Imagery, y: years, M: male, F: female, PSQI: Pittsburg Sleep Quality Index, n: number.

### Functional results

**Figure 1:**
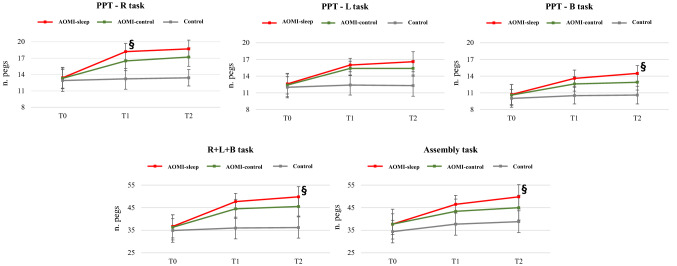
Between-group differences over time for the primary outcome (Purdue Pegboard Test—PPT) are shown (3 × 3 General Linear Model with Bonferroni post-hoc analysis). Data are presented as mean (dots) and standard deviation (bars), and symbols (§) represent significant differences between AOMI-sleep and AOMI-control groups.

The PPT inclusive of right hand (R), left hand (L), both hands (B), R + L + B and assembly tasks and the FTT were performed, and a 3 × 3 General Linear Model was adopted for data analysis (see Material and Methods). Time by Group interactions, Group and Time effects were found for all PPT items (*p* < 0.001 for all interactions and main effects) (Fig. 1). Between-group post-hoc analysis revealed that AOMI-sleep had greater score for R task at T1 (MD: 1.72, CI_95_ 0.12–3.31, p = 0.032, *d* = 1.1) and higher B (MD: 1.6, CI_95_ 0.28–2.92, *p* = 0.013, *d* = 1.1), R + L + B (MD: 4.2, CI_95_ 0.18–8.31, p = 0.038, *d* = 1.0) and assembly (MD: 4.8, CI_95_ 0.07–9.58, p = 0.046, *d* = 0.9) tasks scores at T2, when compared to AOMI-control. Moreover, AOMI-sleep and AOMI-control showed higher R (MD: 4.9, CI_95_ 3.3–6.6, *d* = 2.8 for AOMI-sleep, MD: 3.2, CI_95_ 1.7–4.9, *d* = 1.7 for AOMI-control), L (MD: 3.6, CI_95_ 2.3–5.0, *d* = 2.3 for AOMI-sleep, MD: 3.1, CI_95_ 1.7–4.5, *d* = 1.9 for AOMI-control) B (MD: 2.9, CI_95_ 1.6–4.2, *d* = 2.0 for AOMI-sleep, MD: 2.0, CI_95_ 0.7–3.3, *d* = 1.5 for AOMI-control) R + L + B (MD: 11.7, CI_95_ 7.9–15.6, *d* = 2.7 for AOMI-sleep, MD: 8.5, CI_95_ 4.7–12.4, *d* = 1.8 for AOMI-control) and assembly (MD: 8.6, CI_95_ 4.2–13.1, *d* = 1.9 for AOMI-sleep, MD: 5.5, CI_95_ 1.1–10.0 *d* = 1.1 for AOMI-control) tasks scores compared to Control at T1 (*p* < 0.001 for all comparisons). AOMI-sleep and AOMI-control also revealed higher R (MD: 5.2, CI_95_ 3.7–6.7, *d* = 3.3 for AOMI-sleep, MD: 3.8, CI_95_ 2.2–5.9, *d* = 2.3 for AOMI-control), L (MD: 4.3, CI_95_: 2.7–5.9, *d* = 2.3 for AOMI-sleep, MD: 3.1, CI_95_ 1.5–4.7, *d* = 1.8 for AOMI-control) B (MD: 3.7, CI_95_ 2.4–5.1, *d* = 2.5 for AOMI-sleep, MD: 2.1, CI_95_ 0.8–3.5, *d* = 1.5 for AOMI-control) R + L + B (MD: 13.5, CI_95_ 9.5–17.6, *d* = 2.9 for AOMI-sleep, MD: 9.3, CI_95_ 5.2–13.3, *d* = 2.0 for AOMI-control) and assembly (MD: 10.7, CI_95_ 5.8–15.5, *d* = 2.1 for AOMI-sleep, MD: 5.8, CI_95_ 0.9–10.7, *d* = 1.2 for AOMI-control) tasks scores compared to Control at T2 (*p* < 0.001 for all comparisons). Within-group post-hoc analysis revealed that AOMI-sleep and AOMI-control improved PPT score in all tasks from T0 to T1 (*p* < 0.001 for all comparisons) and T2 (*p* < 0.001 for all comparisons), while Control increased the assembly task score from T0 to T1 (*p* = 0.005) and T2 (*p* < 0.001). AOMI-sleep also increased B (*p* = 0.003) and assembly (*p* < 0.001) tasks scores from T1 to T2. The FTT performed with the right and left hands revealed no Time by Group interactions, Group or Time effects.

### Kinematic and neurophysiological results

The NHPT executed with the right and left hands was performed and kinematic indexes were detected, while a 3 × 3 General Linear Model was used for data analysis (see Material and Methods). The NHPT with the right hand revealed a Time by Group interaction for Peg-In-Hole time, while a Group effect was found for Peg-grasp time, mean velocity during peg-transfer and hand-return, and velocity peak during hand return (Table 2). Between-group post-hoc analysis revealed better Peg-grasp time for AOMI-sleep compared to Control at T2 (*p* = 0.023). In addition, mean velocity during peg-transfer resulted lower for AOMI-sleep compared to Control (*p* = 0.032) at T1. Similarly, mean velocity during peg-transfer and velocity peak during hand-return were lower for AOMI-sleep and AOMI-control, when compared to Control (*p* ≤ 0.001) at T1. Finally, a Time effect was found for Total, Removing, Transfer, Peg-In-Hole and Return times, and mean velocity and velocity peak during peg-transfer and hand-return. Within-group post-hoc analysis in shown in Table 2. The NHPT with the left hand revealed a Group effect for mean velocity during hand-return (Table 3), which resulted lower in AOMI-sleep at T0 (*p* = 0.023) and T1 (*p* = 0.021) and in AOMI-control at T1 (*p* = 0.029) compared to Control, as demonstrated by between-group post-hoc analysis. A Time effect was found for Total, Removing, Transfer, Peg-In-Hole and Return times, normalized jerk and velocity peak during peg-transfer, and for velocity peak during hand-return. Within-group post-hoc analysis in shown in Table 3.

**Table 2:** Between-group differences over time for kinematic indexes during Nine Hole Peg Test performed with the right hand (3 × 3 General Linear Model with Bonferroni post-hoc analysis). Data are shown as mean and standard deviation.

	AOMI-sleep			AOMI-control			Control			*p*-value Time Factor	*p*-value Group Factor	*p*-value Time x Group
	T0	T1	T2	T0	T1	T2	T0	T1	T2			
*Nine Hole Peg Test—Right hand*												
Total time [s]	21.4 ± 2.7	19.0 ± 2.4*	18.5 ± 1.6*	21.9 ± 4.1	18.8 ± 2.1*	18.4 ± 2.6*	21.7 ± 3.4	20.2 ± 2.9	19.5 ± 2.1*	** < 0.001**	0.581	0.433
Removing time [s]	7.8 ± 1.7	6.6 ± 0.9*	6.3 ± 0.7*	7.9 ± 2.5	6.5 ± 1.5*	6.2 ± 1.3*	7.5 ± 1.5	6.8 ± 1.3	6.4 ± 1.1*	** < 0.001**	0.985	0.466
Peg-grasp time [s]	4 ± 0.8	3.7 ± 0.9	3.5 ± 0.5 †	4.2 ± 0.8	3.8 ± 0.8	4 ± 0.9	4.6 ± 1.2	4.4 ± 1.3	4.4 ± 0.8	0.151	**0.021**	0.940
Peg-transfer time [s]	3.3 ± 0.4	3.0 ± 0.5	3.0 ± 0.5	3.1 ± 0.6	2.8 ± 0.4	2.9 ± 0.4	3.4 ± 0.7	3.1 ± 0.6	3.1 ± 0.4	**0.008**	0.104	0.997
Peg-in-hole time [s]	3.4 ± 0.8	2.9 ± 0.5	2.9 ± 0.6	4.1 ± 1.2	3.1 ± 0.7*	2.8 ± 0.6*	3.4 ± 0.8	3.4 ± 0.4	2.9 ± 0.7 α	** < 0.001**	0.427	**0.029**
Hand-return time [s]	2.5 ± 0.3	2.4 ± 0.4	2.3 ± 0.2	2.4 ± 0.3	2.2 ± 0.3	2.2 ± 0.2	2.4 ± 0.5	2.2 ± 0.4	2.3 ± 0.3	**0.004**	0.629	0.951
N-Jerk transfer [m/s^3^]	172.9 ± 39.0	160.4 ± 42.2	164.1 ± 44.0	155.8 ± 58.7	141.6 ± 36.9	147.7 ± 40.1	188.2 ± 71.7	142.6 ± 54.0	164.4 ± 32.1	0.053	0.221	0.618
N-Jerk return [m/s^3^]	134.2 ± 23.7	130.7 ± 36.1	129.0 ± 16.1	124.7 ± 29.1	116.5 ± 27.3	121.4 ± 20.5	133.1 ± 42.5	116.8 ± 34.5	119.9 ± 31.0	0.144	0.436	0.829
Velocity transfer [m/s]	0.28 ± 0.02	0.29 ± 0.03 †	0.29 ± 0.02	0.30 ± 0.04	0.30 ± 0.03	0.30 ± 0.03	0.30 ± 0.03	0.33 ± 0.04*	0.31 ± 0.03	**0.043**	**0.020**	0.308
Velocity return [m/s]	0.36 ± 0.03	0.37 ± 0.04 †	0.37 ± 0.04	0.36 ± 0.05	0.36 ± 0.03 †	0.37 ± 0.02	0.38 ± 0.04	0.42 ± 0.04*	0.40 ± 0.04	**0.013**	**0.003**	0.102
Velocity-peak transfer [m/s]	0.55 ± 0.07	0.60 ± 0.08*	0.60 ± 0.05	0.62 ± 0.08	0.63 ± 0.07	0.64 ± 0.09	0.60 ± 0.07	0.64 ± 0.11	0.63 ± 0.08	**0.012**	0.064	0.676
Velocity-peak return [m/s]	0.77 ± 0.07	0.84 ± 0.07 †	0.84 ± 0.11	0.82 ± 0.11	0.82 ± 0.05 †	0.90 ± 0.09	0.82 ± 0.12	0.93 ± 0.13*	0.86 ± 0.05*	** < 0.001**	**0.028**	0.121

*p < 0.05 compared to T0 for the same group, α *p* < 0.05 compared to T1 for the same group, †*p* < 0.05 compared to Control at the same time-point. AOMI: Action Observation plus Motor Imagery, n: number.

**Table 3:** Between-group differences over time for kinematic indexes during Nine Hole Peg Test performed with the left hand (3 × 3 General Linear Model with Bonferroni post-hoc analysis). Data are shown as mean and standard deviation.

	AOMI-sleep			AOMI-control			Control			*p*-value Time Factor	*p*-value Group Factor	*p*-value Time x Group
	T0	T1	T2	T0	T1	T2	T0	T1	T2			
*Nine Hole Peg Test – Left hand*												
Total time [s]	22.7 ± 2.4	20.4 ± 2.5*	20.0 ± 2.3*	22.9 ± 4.0	21.1 ± 2.7*	20.6 ± 2.8*	22.4 ± 3.4	21.9 ± 3.0	20.4 ± 2.6 *α	** < 0.001**	0.822	0.254
Removing time [s]	7.9 ± 1.6	6.8 ± 0.9*	6.7 ± 1.1*	7.9 ± 2.0	7.0 ± 1.8*	6.6 ± 1.2*	7.9 ± 1.7	7.2 ± 1.5	6.9 ± 1.6*	** < 0.001**	0.955	0.872
Peg-grasp time [s]	4.3 ± 1.0	4.0 ± 1.1	3.9 ± 0.8	4.2 ± 0.8	4.3 ± 0.8	4.4 ± 0.9	4.2 ± 1.1	5.0 ± 1.7	4.2 ± 0.8	0.331	0.402	0.101
Peg-transfer time [s]	3.4 ± 0.3	3.2 ± 0.5	3.2 ± 0.5	3.6 ± 0.7	3.3 ± 0.5	3.3 ± 0.5	3.5 ± 0.9	3.1 ± 0.4	3.3 ± 0.5	**0.004**	0.671	0.893
Peg-in-hole time [s]	4.1 ± 0.5	3.6 ± 1.2	3.4 ± 0.9*	4.4 ± 1.3	3.8 ± 0.8	3.7 ± 0.9	3.9 ± 0.9	3.9 ± 1.0	3.2 ± 0.4 α	** < 0.001**	0.450	0.496
Hand-return time [s]	2.6 ± 0.5	2.4 ± 0.2	2.4 ± 0.3	2.4 ± 0.3	2.3 ± 0.3	2.3 ± 0.2	2.5 ± 0.5	2.3 ± 0.3	2.5 ± 0.4	**0.027**	0.237	0.375
N-Jerk transfer [m/s^3^]	180.2 ± 40.4	170.2 ± 43.0	169.4 ± 37.5	204.7 ± 73.0	169.9 ± 51.1	180.6 ± 47.7	195.5 ± 93.9	154.1 ± 39.8	174.6 ± 42.0	**0.018**	0.667	0.746
N-Jerk return [m/s^3^]	150.5 ± 60.0	130.9 ± 21.9	128.9 ± 23.1	121.7 ± 22.3	118.1 ± 26.4	111.5 ± 19.5	134.3 ± 48.2	120.3 ± 27.8	132.9 ± 31.9	0.079	0.120	0.438
Velocity transfer [m/s]	0.27 ± 0.02	0.29 ± 0.03	0.30 ± 0.02*	0.27 ± 0.04	0.28 ± 0.04	0.29 ± 0.04	0.30 ± 0.04	0.31 ± 0.05	0.30 ± 0.04	0.004	0.111	0.307
Velocity return [m/s]	0.35 ± 0.04 †	0.37 ± 0.03 †	0.37 ± 0.04	0.37 ± 0.03	0.37 ± 0.02 †	0.40 ± 0.04	0.39 ± 0.04	0.40 ± 0.05	0.38 ± 0.04	0.022	**0.023**	0.058
Velocity-peak transfer [m/s]	0.54 ± 0.04	0.59 ± 0.06	0.61 ± 0.05*	0.54 ± 0.11	0.57 ± 0.08	0.58 ± 0.09	0.60 ± 0.11	0.61 ± 0.11	0.59 ± 0.08	**0.019**	0.410	0.344
Velocity-peak return [m/s]	0.75 ± 0.09	0.82 ± 0.10	0.84 ± 0.11	0.82 ± 0.09	0.85 ± 0.07	0.90 ± 0.05	0.85 ± 0.15	0.91 ± 0.15	0.87 ± 0.07	**0.003**	0.070	0.191

*p < 0.05 compared to T0 for the same group, α *p* < 0.05 compared to T1 for the same group, †*p* < 0.05 compared to Control at the same time-point. AOMI: Action Observation plus Motor Imagery, n: number.

**Figure 2:**
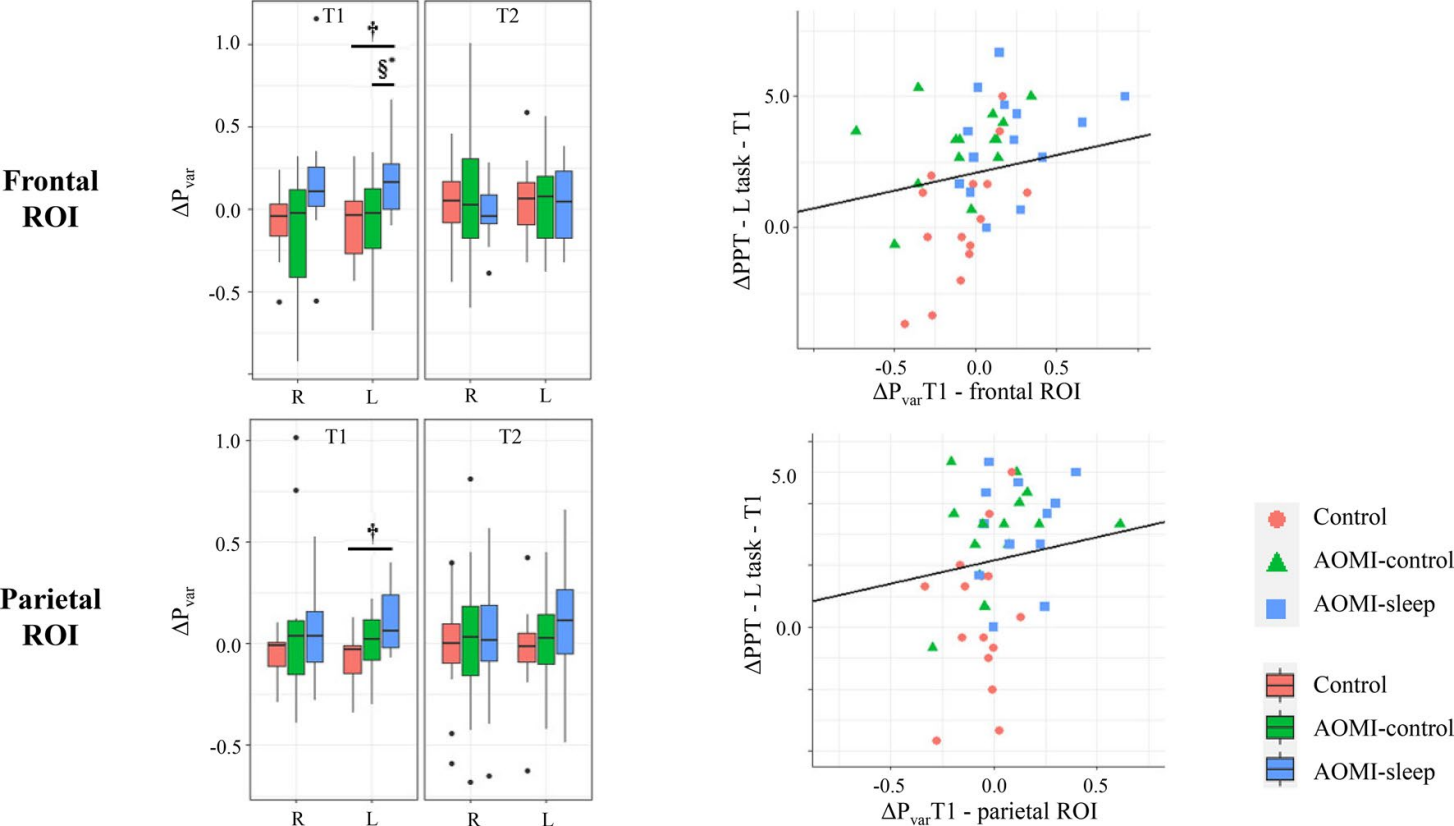
Between-group differences for ΔP_var_T1 and ΔP_var_T2 of mu rhythm in the frontal and parietal ROIs during NHPT with the right (R) and left (L) hands. Boxes represent the range between the first and the third quartile, the middle horizontal line is the mean value, and the end of the vertical line are the maximum and minimum values (outliers are also shown). Symbols show AOMI-sleep differences with AOMI-control (**§)** and Control (**†)**. Positive correlation between ΔP_var_T1 of the frontal and parietal ROIs during NHPT with the left hand and changes in the left task of the PPT from T0 to T1 (ΔPPT—L task—T1).

Electroencephalographic signals were also recorded during NHPT execution to detect power variations (P_var_) in the mu band with respect to resting state with eyes open in frontal, central and parietal regions of interest (ROIs). The topographical map of the median P_var_ in the mu band for all participants during the NHPT performance at each timepoint is shown in the supplementary material 2. P_var_ changes from T0 to T1 (ΔP_var_T1) and T2 (ΔP_var_T2) were computed for each ROI and compared between the three groups using a Univariate ANOVA model. No Group effects were found for ΔP_var_T1 and ΔP_var_T2 of frontal, central and parietal ROIs during the NHPT with the right hand (Supplementary material 3). A Group effect was found for ΔP_var_T1 at the level of the frontal (*p* = 0.012) and parietal (*p* = 0.049) ROIs during the NHPT with the left hand. Post-hoc analysis revealed higher ΔP_var_T1 for AOMI-sleep compared to AOMI-control (*p* = 0.016) and Control (*p* = 0.012) for the frontal ROI, while higher ΔP_var_T1 was found for AOMI-sleep compared to Control (*p* = 0.023) for the parietal ROI (Fig. 2 and Supplementary material 3). On the other hand, no Group effects were found for ΔP_var_T1 of central ROI and for ΔP_var_T2 of frontal, central and parietal ROIs (Supplementary material 3). Finally, Pearson’s correlation coefficients revealed a moderate positive correlation between changes from T0 to T1 in the L task of the PPT and ΔP_var_T1 of the frontal (r = 0.458, *p* = 0.001) and parietal (r = 0.360, *p* = 0.016) ROIs during NHPT with the left hand (Fig. 2).

## Discussion

The main finding of the study was that AO + MI-training enhanced motor learning in terms of manual dexterity, especially when the training sessions were followed by early sleep. Specifically, participants undergoing AO + MI-training improved manual dexterity, but subjects performing AO + MI-training followed by early sleep revealed significantly larger improvements than those undergoing the same training at least 12 h before sleeping. These behavioral findings were supported by neurodynamical correlates during motor performance. Furthermore, sleep-dependent benefits were also detected at 1 month after the training end.

Our findings agree with studies showing a role of sleep on motor skills acquisition and proved sleep benefits on motor learning, also when the training is delivered through the systematic observation and mental practice of motor tasks in the absence of execution^25^. Sleep-dependent gains in motor performance after a training have been reported to occur through an offline replay of neural activity, able to induce a memory consolidation of newly acquired movement patterns^21^. This reactivation process is documented by specific EEG markers (e.g., sleep spindles) and involves the cortico-striato-cerebellar network, where the degree of reactivation seems to be related to ameliorations in terms of motor performance^23^. Previous studies demonstrated benefits induced by AO/AO + MI in terms of manual dexterity^13,15,27,28^. However, the timing of AO + MI-training administration proposed in the current study seems to further enhance the training effects, leading us to account larger manual dexterity improvements experienced by AOMI-sleep group to a motor learning consolidation process induced by early sleep^18,19^. AO + MI-training efficacy has been described as based on the development of an internal model of human movements through observation and imagination of actions performed by others, which allow for the formation of a motor memory^2^. Our results suggested that the refinement of the internal model triggered by AO + MI-training may continue after the training session, where a sleep period seems to increase the resistance of the model traces against the behavioral interference^29^. Furthermore, studies reported that the retrograde interference plays a role on motor learning, revealing that the acquisition of new memory traces decreases the consolidation of previously acquired information^30,31^. When considering the additional sleep-dependent benefits detected in favor of AOMI-sleep group, it is reasonable to speculate that the consolidation process of newly acquired motor skills might have been less affected by retrograde interference when memory traces were acquired before sleeping. Conversely, daily activities experienced by AOMI-control group after training sessions might have weakened the consolidation process of motor skills acquired through AO and MI.

When considering our study design in relation to obtained results, the potential influence of circadian rhythms on motor imagery ability deserves to be also considered. In particular, literature data adopted the temporal equivalence between imagined and executed movements as indicator of motor imagery accuracy, reporting that the ability of healthy subjects to internally simulate their own movements fluctuated through the day^32^. However, Gueugneau and co-workers exclusively detected such temporal equivalence in the afternoon between 2:00 p.m. and 8:00 p.m.^33,34^. Therefore, it is reasonable to speculate that this aspect might have not affected our results, since AO + MI-training was performed in the morning or during the evening, where, despite a lack of isochrony, similar temporal discrepancy between executed and imagined movements was observed by previous studies^33,34^.

In the current study, greater enhancement in manual dexterity for AOMI-sleep group occurred at the level of the trained limb, similarly to previous studies showing a task-specificity of motor learning induced by observation in healthy subjects^6,13,25^. The exploitation of the mirror mechanism for the acquisition of new motor skills has been reported to induce behavioral modifications accompanied by structural and functional brain changes in neural networks involved in movements performed with trained body segments^13^. Nevertheless, our findings revealed that larger sleep-dependent benefits also occurred during the performance of bimanual complex tasks (e.g., B and assembly PPT tasks). These additional improvements were detected at 1-month follow-up in the AOMI-sleep group, demonstrating not only the occurrence of enduring behavioral changes, but also a further optimization of the acquired motor skills^35^. Lugassy and co-workers demonstrated that a sleep-dependent consolidation of complex motor skills mainly occurred into a time-window longer than 24 h after the practice end^36^. Our findings showed that similar trajectories of motor learning can be identified also when complex motor skills are trained through AO + MI. In this scenario, the occurrence of such long-term benefits agrees with previous studies adopting AO and MI to improve motor performance and contributes to prove the relevance of these approaches to enhance the consolidation of motor learning^37,38^.

The kinematic assessment revealed manual dexterity improvements induced by AO + MI-training, which occurred at training end and persisted up to 1 month in AOMI-sleep and AOMI-control groups with no differences between the two groups. Improvements mainly occurred for total and removing NHPT times and during subphases requiring higher finger dexterity, such as peg-grasp and peg-in-hole. Similarly to functional results, these ameliorations were detected bilaterally. Improvements at the level of the untrained hand agree with studies showing an interlimb transfer effect from the dominant to non-dominant hand after a unilateral dexterity training^39^. In addition, studies reported bilateral MNS activity during observation and imagination of unilateral tasks, leading to bilateral functional changes in frontoparietal brain areas after AO + MI interventions^40^. In this light, untrained limb improvements experienced by study participants may be accounted to an interlimb transfer effect induced by AO + MI-training. Coherently, higher frontoparietal cortical activation was detected in AOMI groups during NHPT performance with the left hand. On the other hand, it is worth noting that lower velocity was found during peg-transfer and hand-return subphases of the NHPT in subjects undergoing AO + MI-training, when compared to Control group. However, it is reasonable to speculate that greater reliance on sensorimotor network during upper limb movements may resulted in slower but highly controlled trajectories for AOMI groups^41^.

As anticipated, the opportunity to further enhance manual dexterity through immediate sleep after AO + MI-training was also supported by EEG results, showing greater mu rhythm desynchronization in frontal and parietal brain areas in AOMI-sleep group during NHPT with the left hand at the training end. The magnitude of mu rhythm suppression represents an index of sensorimotor system activation during movements planning and execution^42^. Increased mu rhythm suppression has been described as a result of increased excitability of cortical regions occurring in the presence of motor learning, which can be enhanced by exploiting the MNS peculiarities through AO + MI-training^43^. Therefore, our findings may be interpreted as an increase in top-down control during a complex dexterity task requiring marked sensorimotor abilities in subjects undergoing immediate sleep after AO + MI-training. The current brain functional changes were detected in the early phase after training end, when cortical plasticity processes related to motor learning are usually more vivid^44,45^. In fact, cortical activity returned similar to baseline levels at 1-month follow-up, although no changes in NHPT performance occurred. Furthermore, positive correlation between changes in frontal and parietal mu rhythm desynchronizations during left NHPT and variations in the PPT task performed with the left hand suggested that higher frontoparietal activation was associated to improvements in manual dexterity. Consistently with behavioral findings, the left hand consisted of the untrained side, which may be passive of higher manual dexterity gains through an interlimb transfer effect.

Some limitations of the current study need to be underlined. First, imagination abilities and the chronotype of participants were not investigated though specific outcome tools. However, the study had randomized design and these variables are expected to be equally distributed. Second, although participants’ adherence was monitored using daily message on a smartphone and a diary, AO + MIinterventions were unsupervised, and fluctuations of the attentive status might have influenced the training effects. Finally, sleep quantity and quality were monitored using the self-reported number of hours and a specific questionnaire. However, physiological investigations might have provided better sleep monitoring and neurophysiological correlates of sleep-dependent learning induced by AO + MI-training.

## Conclusions

Additional AO + MI-training benefits in terms of manual dexterity were found when the training sessions were followed by immediate sleep. These findings proved a sleep dependence of motor learning induced by AO + MI-training and may be crucial in patients with upper limb impairments, where conventional rehabilitation delivered in association with AO + MI-training followed by early sleep may facilitate motor and functional recovery.

## Methods

### Participants

Forty-five healthy volunteers were enrolled between December 2020 and December 2021. Participants aged between 18 and 30 years old and reported the right hand as dominant according to the Edinburgh Handedness Inventory^46^. Exclusion criteria were history of musculoskeletal or neurological conditions affecting upper limb function, or practice of sports, jobs or activities requiring advanced manual skills. Subjects with documented sleep disorders (e.g., insomnia, obstructive sleep apnea syndrome, REM or non-REM behavior disorders) or reporting the use of medications able to influence the physiological sleep pattern were also excluded. Participants’ characteristics are reported in the Table 1. The study was performed at the Motion and Posture Analysis Lab and Biosignal, Bioimage, Biodata Lab of Politecnico di Milano, Italy. All methods were performed in accordance with the relevant guidelines and regulations, a written informed consent was obtained from all subjects or their legal guardians, and the study protocol was approved by the Humanitas Clinical and Research Center Ethical Committee (n. CLF20/08, July 2020). Informed consent from participants or their legal guardians for publication of identifying information/images in an online open-access publication was also obtained.

### Study design

The study has a three-armed single-blind randomized controlled design. After the enrollment, participants were randomized into AOMI-sleep (n = 15), AOMI-control (n = 15) or Control (n = 15) groups using a simple computer-generated sequence. An independent researcher assigned participants to training groups according to the randomization list in order to ensure the allocation concealment.

All participants were asked to watch 20 min. video-clips in seated posture on a 15-inch computer screen, 4 days a week per 3 weeks. Participants allocated in AOMI-sleep and AOMI-control groups were asked to watch video-clips representing transitive daily tasks performed with the right upper limb and requiring advanced manual skills (Supplementary material 1). Both groups received the same visual stimuli, and the only difference was that subjects allocated to AOMI-sleep group underwent the training between 8:00 and 10:00 p.m., whereas participants included in AOMI-control group performed the training between 8:00 and 10:00 a.m. Video-clips included four tasks (5 min per task) delivered in third (2 min per task) and first-person (3 min per task) perspectives. Actors’ gender was congruent with those of the observers in order to enhance empathy, and first-person observation was concurrently associated with motor imagery of the observed task, as recommended by Romano Smith and co-workers^11,47^. Precisely, participants were requested to carefully observe video-clips staying as still as possible and focusing on how the tasks were performed. In addition, during first-person perspective observation, they were asked to imagine themselves performing the movement as they would actually execute and focus on sensations caused by actual movements (kinesthetic motor imagery). Participants were explicitly asked to practice no movements during observation and imagination and the complexity of the proposed tasks progressively increased over the 3 weeks. Subjects allocated to control group were asked to watch video-clips representing landscapes without motor contents between 8:00 and 10:00 p.m. During the 3 weeks of training, participants were asked to avoid daytime sleep, and adherence to the treatment was ensured through a daily message on the smartphone. In addition, participants were asked to fill in a diary reporting the time in which the training sessions were performed and the number of nightly sleep hours in the training period. Finally, participants’ sleep quality in the training period was assessed through the administration of the Pittsburg Sleep Quality Index (PSQI) at the end of training^48^.

### Functional, kinematic and neurophysiological assessment

All participants underwent a functional, kinematic and neurophysiological assessment supervised by an operator blinded to group allocation. Data were collected at baseline (T0), at the training end (T1) and at 1 month after the training end (T2).

Functional assessment included the Purdue Pegboard Test (PPT) as primary outcome and the Finger Tapping Test (FTT). The PPT consists of a rectangular board with two lines of holes and four cups containing pegs, collars, and washers. Participants were asked to perform three subtests in which they had to insert pegs in the holes with the right (R), left (L) and both hands simultaneously (B), and one assembly task in which they had to assemble pegs, collars and washers using alternate hand movements. The tasks had to be performed as quickly as possible, and participants had 30 s for R, L and B tasks, whereas 60 s were assigned to accomplish the assembly task. The number of inserted pins represents the score, and higher score indicates better manual dexterity. Three trials were attempted, and the mean score was calculated^49^. During the FFT, participants were asked to seat on a chair with the forearms resting on a desk in front of them and tap a counter button with the index fingers of the right and left hands as rapidly as possible for 10 s. Five trials interspaced by a 30 s rest were performed, and the mean score was computed^50^.

Kinematic assessment included the Nine Hole Peg Test (NHPT) and the detection of kinematic indexes during the performance^51^. The NHPT was performed with the participants seated on a height-adjustable chair without back support with the pegboard positioned in front of participants on a 70-cm high table. They were asked to grasp nine pegs from a container, insert them into a nine-hole grid, and replace the pegs into the container as quickly as possible. The performance was recorded using an optoelectronic system (SMART-DX, BTS, Italy) equipped with eight infrared cameras. Three retro-reflective markers were placed on the left and right acromial angles and on the jugular incisura to identify the trunk, two markers were placed on the left and right middle phalanx of the index finger to trace hand trajectories, while three markers were placed on the left distal and left and right proximal corners of the table to define the global reference system. Raw marker data were sampled at 100 Hz and filtered using a fourth-order low-pass Butterworth filter (cut-off 4 Hz). Subsequently, the following kinematic indexes were computed during the NHPT execution using the procedures and algorithm described by Temporiti and co-workers: total and single phases times (peg-grasp, peg-transfer, peg-in-hole, hand-return), and normalized jerk, mean and peak of velocity during peg-grasp and hand-return phases^51^. These indexes during the NHPT represent valid and reliable parameters for manual dexterity assessment in young healthy subjects. After a familiarization trial, two trials for each side were performed, and the shortest trial was considered for data analysis.

Neurophysiological assessment included electroencephalographic (EEG) signals recording during the NHPT. A 64-channel continuous EEG system (SD LTM 64 Express, Micromed, Italy) with Ag/AgCl surface electrodes mounted on a cap according to the Standard International 10/20 system was used to acquire brain signals, sampled at 1024 Hz^52^. The ground electrode had a mid-forehead placement (between Pz and CPz positions) and impedances were kept lower than 20kOhm. Bipolar surface electromyography Ag/AgCl electrodes were also placed bilaterally on the anterior deltoid according to SENIAM recommendations to synchronize EEG tracks with the optoelectronic system through the detection of neuromuscular activity onset^53^. Before the NHPT performance, a 1 min eyes-open resting period was acquired. Data were imported in MATLAB and pre-processed using EEGLAB^54^. First, signals were band-pass filtered in the 1–45 frequency range using a FIR zero-phase filter and down-sampled at 256 Hz. Subsequently, artifacts were removed using Independent Component Analysis, and Common Average Re-referencing was applied. The analysis was focused on EEG spectral profile of the mu rhythm, being a reliable indicator and electrophysiological correlate of sensorimotor system activation during movement planning and execution^42,55^. The individual mu rhythm was defined as the 2-Hz frequency range centered around the individual alpha frequency (IAF) of the motor cortex, identified as the frequency in the range 7–13 Hz showing the highest activity during movement execution with respect to the resting period. Specifically, the IAF was the average between the IAFs at C3 and C4 locations^56,57^. 

Cortical activation changes during motor performance were quantified for each electrode in terms of power variation (P_var_) compared to the resting condition, according to the formula:

$$ P_{{{\text{var}}}} = \frac{{P_{{{\text{mov}}}} - P_{{{\text{rest}}}} }}{{P_{{{\text{rest}}}} }} $$

where, P_mov_ represents the power observed in the mu frequency band during motor execution, and P_rest_ is the power of the same frequency band during the resting phase with eyes open. Three regions of interests (ROIs) were identified by averaging P_var_ for the following electrode clusters: frontal (Fp1, Fp2, Af7, Af3, Af4, Af8, F7, F5, F3, F1, F2, F4, F6, F8) central (Fc2, Fc4, C2, C4, C6, Cp4, Cp6, Fc1, Fc3, C1, C3, C5, Cp1, Cp3) and parietal (P3, Pz, P4, P5, P1, P2, P6). Finally, changes in terms of P_var_ from T0 to T1 (ΔP_var_T1) and T2 (ΔP_var_T2) were computed for each ROI. Positive ΔP_var_ values indicate greater desynchronization at T1 and T2 compared to T0.

### Statistical analysis

Sample size was estimated a-priori based on the R task of the PPT (primary outcome). It was estimated that, considering a two-tailed alpha error of 5%, a minimum of 15 participants were required in each group to provide 80% power to detect a Cohen’s *d* = 1.0 (large effect size) between AOMI-sleep and AOMI-control groups at T1^58^.

Data were checked for normality using the Shapiro–Wilk test and described as mean and standard deviation. Univariate Analysis of Variance (ANOVA) or Chi-Square test were used to assess between-group differences for participants’ characteristics at T0, PSQI at T1 and sleep hours per night during the training period. A 3 × 3 General Linear Model with Time as within-subjects variable and Group as between-subjects variable was used to investigate between-group differences over time in terms of functional and kinematic outcomes. In the case of significant interactions or main effects, Bonferroni post-hoc tests were used to investigate between-group differences at each time-point and within-group differences among the three time-points. The effect size between the three groups was computed for the primary outcome and expressed as mean difference (MD) with 95% confidence interval (CI_95_) and Cohen’s *d.* Cohen’s *d* was interpreted as small (between 0.2 and 0.5), medium (between 0.5 and 0.8), large (between 0.8 and 1.3) and very large (greater than 1.3)^58^. Finally, Univariate ANOVA with Bonferroni post-hoc tests were performed to investigate between-group differences in terms of ΔP_var_T1 and ΔP_var_T2 of mu rhythm in the frontal, motor and parietal ROIs. Correlations between changes in primary outcome (ΔPPT at T1 and T2) and ΔP_var_T1 and ΔP_var_T2 were also assessed using the Pearson’s correlation coefficient. The strength of correlation was interpreted as small (r-score lower than 0.3), moderate (r-score between 0.3 and 0.6) and strong (r-score greater than 0.6)^58^. Statistical analysis was performed using SPSS 28.0 for Windows and the level of statistical significance was set at α = 0.05.

## Supplementary Information

Supplementary Information.

## Data Availability

The dataset of the current study is available from the corresponding author on reasonable request.
