# Supplementary material for: Early sleep after action observation and motor imagery training boosts improvements in manual dexterity
Source: Sci Rep. 2023 Feb 14;13:2609. doi: 10.1038/s41598-023-29820-5 (PMC9929332; doi:10.1038/s41598-023-29820-5)
Supplement: Supplementary file 1 — Supplementary Information. [file 41598_2023_29820_MOESM1_ESM.docx]

**Early sleep after action observation and motor imagery training boosts improvements in manual dexterity**

**Authors**: Federico Temporiti, Alessandra Calcagno, Stefania Coelli, Giorgia Marino, Roberto Gatti, Anna Maria Bianchi, Manuela Galli

**Supplementary material 1**. Description of video-clips motor contents administered to AOMI-sleep and AOMI-control groups during the 3-week training.

**Week 1**


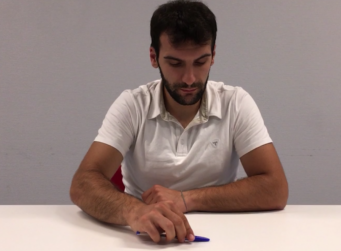

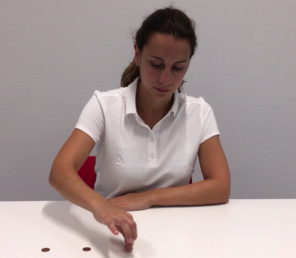

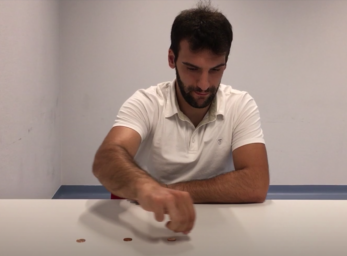

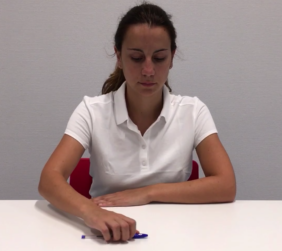

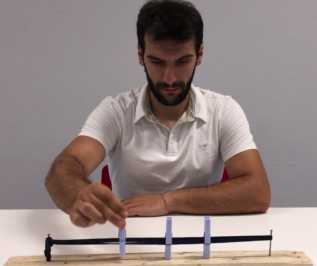

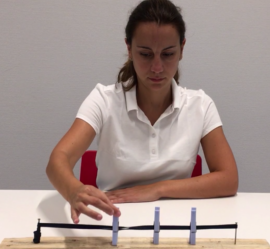

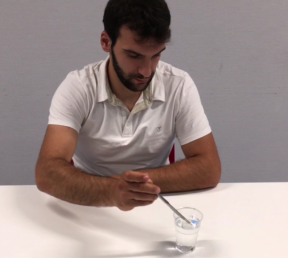


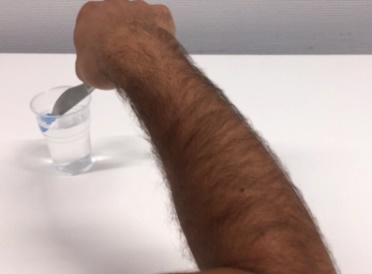

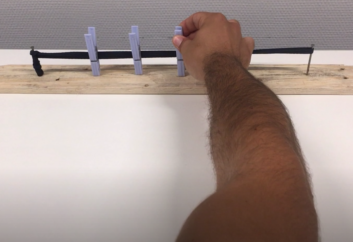
**
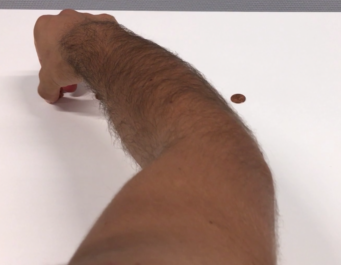
**
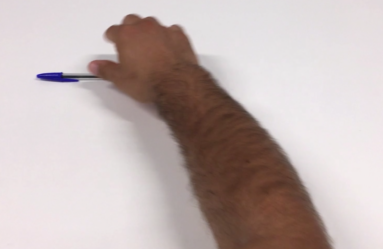


Stacking coins

Removing the cap

from a pen

Hanging

clothespins


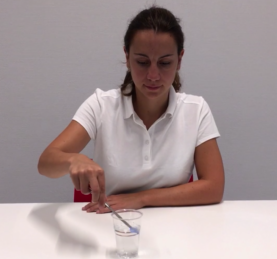


Removing a piece

of paper from a

glass using a knife

**Week 2**

*
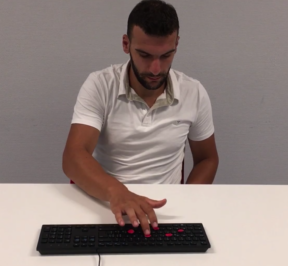

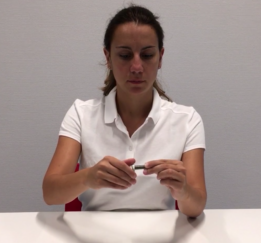

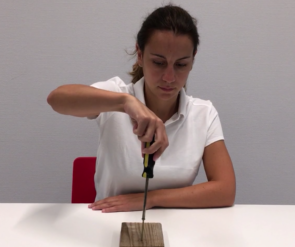
*
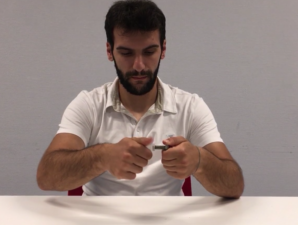

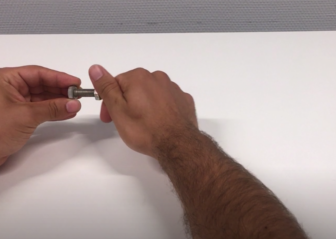

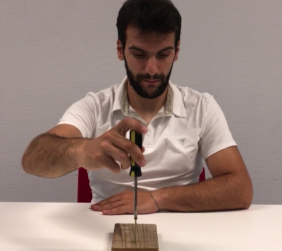

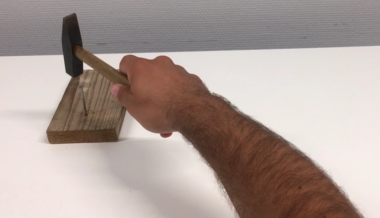

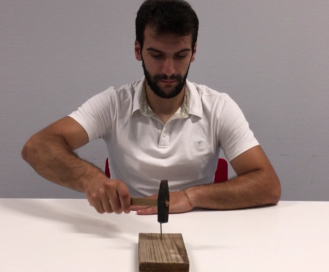

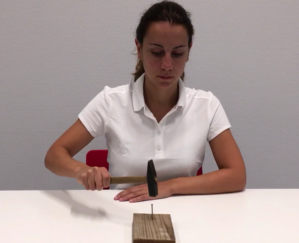


*
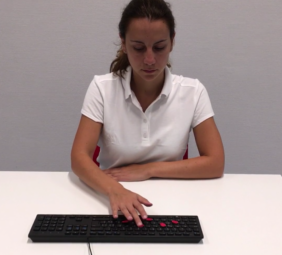
*

Hammering

a nail

Screwing in

a bolt


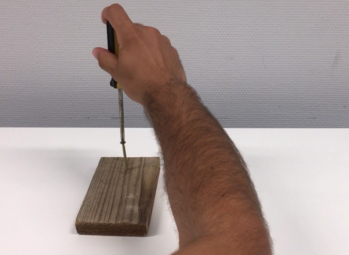

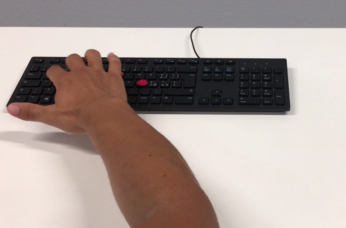


Screwing in a

screw with a

screwdriver

Typing on a

keyboard

**Week 3**


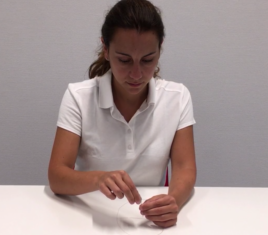

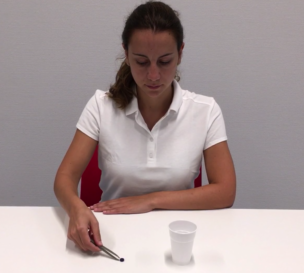

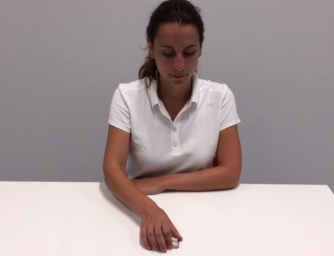

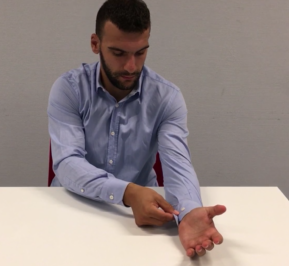

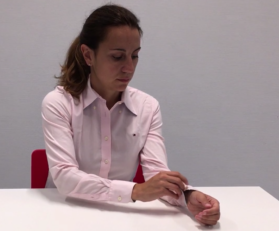

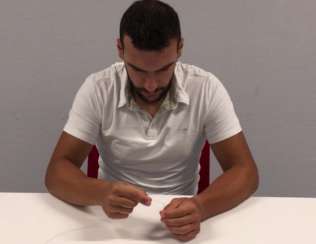

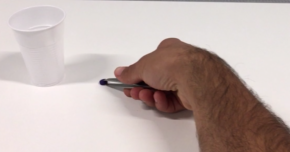

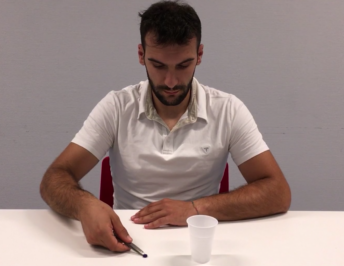

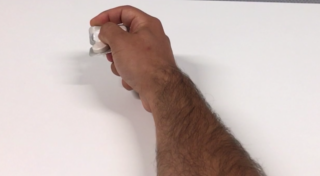

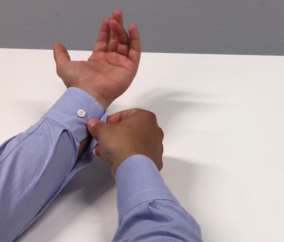

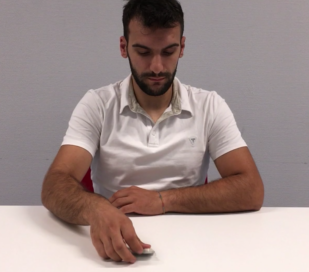


Buttoning up the

cuff of a shirt

Removing a pill

from its packaging

Picking up a pill

With tweezers and

putting it into a glass


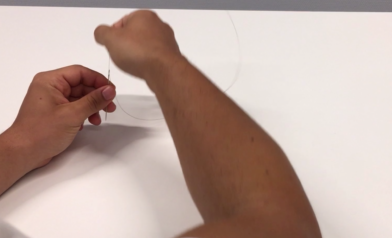


Threading a thread

through the eye

of a needle

**Supplementary material 2.** Topographical map of the median P_var_ in the mu band for all participants at baseline (T0), training end (T1) and at 1-month after the training end (T2) during the Nine Hole Peg Test (NHPT) performed with the right (R-NHPT) and left (L-NHPT) hands. Mu rhythm desynchronization (P_var_ <0) can be observed during NHPT execution especially at the level of central electrodes.


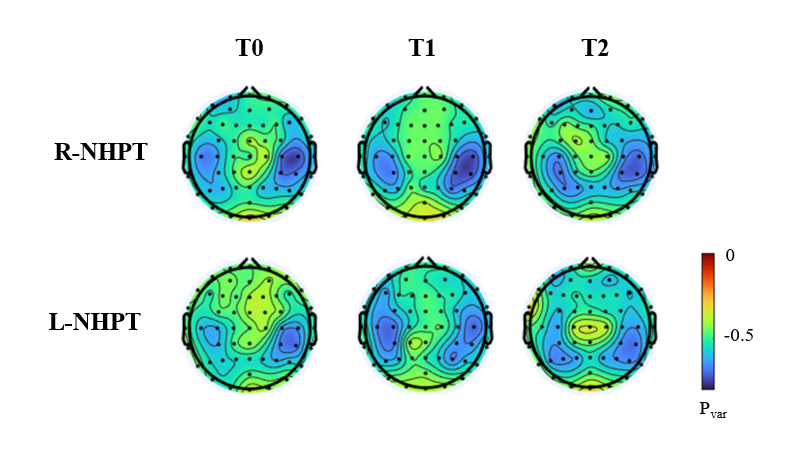


**Supplementary material 3**. Between-group differences over time for ΔP_var_T1 and ΔP_var_T2 of mu rhythm in the frontal, central and parietal ROIs during Nine Hole Peg Test performed with the right and left hands (Univariate ANOVA test with Bonferroni post-hoc analysis). Data are shown as mean and standard deviation.

| **ΔPvar - ROIs** | **AOMI-sleep** | **AOMI-control** | **Control** | **p-value** |  |
| --- | --- | --- | --- | --- | --- |
| *Right hand* | | | | | |
| **ΔPvarT1- Frontal** | 0.14±0.36 | -0.13±0.35 | -0.08±0.19 | 0.080 |  |
| **ΔPvarT1 - Central** | 0.06±0.23 | -0.06±0.24 | -0.05±0.11 | 0.390 |  |
| **ΔPvarT1 - Parietal** | 0.06±0.22 | 0.05±0.37 | -0.05±0.10 | 0.740 |  |
| **ΔPvarT2 - Frontal** | -0.02±0.16 | 0.089±0.43 | 0.03±0.25 | 1.000 |  |
| **ΔPvarT2 - Central** | -0.02±0.28 | 0.00±0.24 | -0.01±0.19 | 1.000 |  |
| **ΔPvarT2 - Parietal** | 0.01±0.33 | 0.03±0.37 | -0.02±0.26 | 1.000 |  |
| *Left hand* | | | | |  |
| **ΔPvarT1- Frontal** | 0.21±0.28 **^†§^** | -0.08±0.29 | -0.07±0.21 | **0.012** |  |
| **ΔPvarT1 - Central** | 0.12±0.23 | -0.02±0.20 | -0.06±0.11 | 0.070 |  |
| **ΔPvarT1 - Parietal** | 0.10±0.15 **^†^** | 0.02±0.21 | -0.06±0.12 | **0.049** |  |
| **ΔPvarT2 - Frontal** | 0.04±0.24 | 0.03±0.26 | 0.04±0.23 | 1.000 |  |
| **ΔPvarT2 - Central** | 0.03±0.31 | -0.00±0.21 | -0.04± 0.21 | 1.000 |  |
| **ΔPvarT2 - Parietal** | 0.08±0.30 | 0.02±0.24 | -0.03±0.22 | 0.920 |  |

**Symbols:** **†** p<0.05 compared to Control, **§** p<0.05 compared to AOT-Control. **Abbreviations**: **AOMI**: Action Observation plus Motor Imagery, **n**: number.
